# Genome Sequences of Three Highly Copper-Resistant *Salmonella enterica* subsp. I Serovar Typhimurium Strains Isolated from Pigs in Denmark

**DOI:** 10.1128/genomeA.01334-14

**Published:** 2014-12-24

**Authors:** Yanan Qin, Henrik Hasman, Frank M. Aarestrup, Hend A. Alwathnani, Christopher Rensing

**Affiliations:** a Department of Plant and Environmental Science, University of Copenhagen, Frederiksberg, Denmark; b National Food Institute, Technical University of Denmark, Lyngby, Denmark; c Department of Botany and Microbiology, King Saud University, Riyadh, Saudi Arabia; d Institute of Urban Environment, Chinese Academy of Sciences, Xiamen, China

## Abstract

*Salmonella typhimurium* is the causative agent of typhoid fever, which causes nearly 21.7 million illnesses and 217,000 deaths around the world each year. Here, we describe the draft genome sequences of the *Salmonella typhimurium* strains S7, S15, and S23, isolated from copper-fed pigs in Denmark and containing additional putative determinants conferring resistances to copper and other metals and metalloids.

## GENOME ANNOUNCEMENT

*Salmonella enterica* subsp. I serovar Typhimurium (*S. typhimurium*) is one of the most important food-borne bacterial pathogens, with a broad host range, including food animals and humans, infecting 21.7 million people and causing 217,000 deaths annually (1–3). In addition, the magnitude of multidrug resistance in *Salmonella* and other pathogens at the human-animal and ecosystem interface has been a major concern globally.

Three *S. typhimurium* (S7, S15, S23) strains were isolated from copper-fed pigs as part of the Danish Integrated Antimicrobial Resistance Monitoring (DANMAP) surveillance program (4). Genomic DNA (gDNA) was purified from the isolates using the Easy-DNA extraction kit (Invitrogen), and DNA concentrations were determined using the Qubit dsDNA BR assay kit (Invitrogen). Whole-genome shotgun sequencing of *S. typhimurium* strains S7, S15, and S23 was performed using the Illumina MiSeq platform to generate 12,037,844, 12,039,576, and 12,061,925 paired-end reads, resulting in ~165-fold, ~170-fold, and ~168-fold coverage of the genomes, respectively. Genome assemblies were constructed *de novo* using CLCBio Genomic Workbench version 5.1 (5) (CLCBio,Denmark), which generated 146, 148, and 148 contigs, respectively.

The resulting contigs were uploaded into the RAST server (6,7) to predict the open reading frames (ORFs). In brief, the predicted ORFs were annotated by searching against clusters of SEED (8) databases. The draft genome size of *S. typhimurium* S7 is approximately 4,927,534 bp in length, with an average GC content of 52.0% and comprises 4,907 predicted coding sequences with an average length of 810 bp. *S. typhimurium* S15 is 4,914,811 bp in length, with an average GC content of 52.1%, and comprises 4,898 predicted coding sequences with an average length of 847 bp. *S. typhimurium* S23 is 4,914,318 bp in length, with an average GC content of 52.1%, and comprises 4,908 predicted coding sequences with an average length of 803 bp.

The three *S. typhimurium* genomes contain several copper-resistance genes, including the Cu(I)-translocating P-type ATPase CopA, multicopper oxidase CueO, and a copper-responsive MerR-family transcriptional regulator CueR (9–11), which were also reported in *S. typhi* strains Ty2 and CT18 (12–14). All three strains contained a mobile 20-gene copper resistance determinant that contained the previously described *pco* and *sil* determinants (15, 16). There are two additional genes between these two determinants; *pcoG* encodes a putative M23B metallopeptidase, and *pcoF* encodes a putative copper binding protein.

The sequences also revealed genes encoding the *Salmonella*-specific P-type ATPase, GolT, *golBS*, and *gesABC* (17, 18, 19) genes. Most *Salmonella* strains lack an RND-type CusCFBA system; instead, CueP sequesters copper to neutralize toxicity (20–23). However, *S. typhimurium* (S7, S15, S23) contained both an RND type system and CueP. In addition, the genome also revealed a six-gene cluster *merEDAPTR* encoding proteins conferring mercury resistance and the five-gene *ars* operon *arsRDABC* (24).

### Nucleotide sequence accession numbers.

These whole-genome shotgun projects have been deposited in DDBJ/EMBL/GenBank under the accession numbers JRGS00000000, JRGR00000000, JRGT00000000. The version described in this paper is the first version. The BioProject designations for those projects are PRJNA260777, PRJNA260771, and PRJNA260778.
